# Two Cases with Incidental Finding of Large Asymptomatic Intradural Lumbar Tumors

**DOI:** 10.7759/cureus.3446

**Published:** 2018-10-12

**Authors:** Jason Hartman, Michelle Granville, Robert E Jacobson

**Affiliations:** 1 Pain Medicine, Larkin Hospital, Miami, USA; 2 Neurological Surgery, University of Miami Hospital, Miami, USA

**Keywords:** lumbar intradural tumors, incidental mri findings, incidental tumors

## Abstract

Benign lumbar intradural tumors are statistically uncommon and usually present with complaints of back pain with or without radicular neurological complaints. This report involves two separate patients that were found incidentally to have large intradural tumors without any neurologic complaints. In both cases the tumors were discovered when having magnetic resonance imaging (MRI) after minor auto accidents. Neither patient had any pre-existing lumbar or neurologic complaints. The report will review the different regions and types of incidental findings commonly seen on lumbar MRI scans and the need for close follow-up in patients with incidental lesions such as tumors.

## Introduction

Incidental radiologic findings are common and detected frequently with the use of higher definition studies such as computerized tomography (CT) and magnetic resonance imaging (MRI) [1,2]. Lumbar MRI and CT studies are most commonly performed for lumbar pain either due to trauma, accidents or spinal degenerative disease, so both clinical and radiographic attention is usually focused on the intervertebral discs and facet joints. However, incidental findings are very common in the lumbar spine as well as the relatively large adjacent abdominal and retro-peritoneal area [3]. These incidental radiologic findings found with studies of the lumbar spine are classified into extraspinal and spinal. Extraspinal findings include abnormalities mainly involving abdominal and retroperitoneal structures including unsuspected renal cysts and pararenal masses, retroperitoneal masses or vascular lesions such as aortic aneurysms. Incidental spinal lesions not related to the original reason for the radiologic study being performed are further classified as vertebral or intraspinal. Unsuspected lesions within the vertebrae are commonly hemangiomas, osteoporotic fractures at the lumbar and lumbar-thoracic levels and bony and metastatic tumors [3-5]. Intraspinal lesions include Tarlov cysts, lipomas, tethered cord and rarely intraspinal tumors as in these two cases. Certain lesions such as suspected metastatic vertebral lesions, unsuspected osteoporotic fractures and rarely intra-vertebral or intraspinal tumors must be identified as in these two cases. Such lesions require more specific follow-up, clear notification to the referring physician and treatment [4-6].

## Case presentation

Case 1

**Figure 1:**
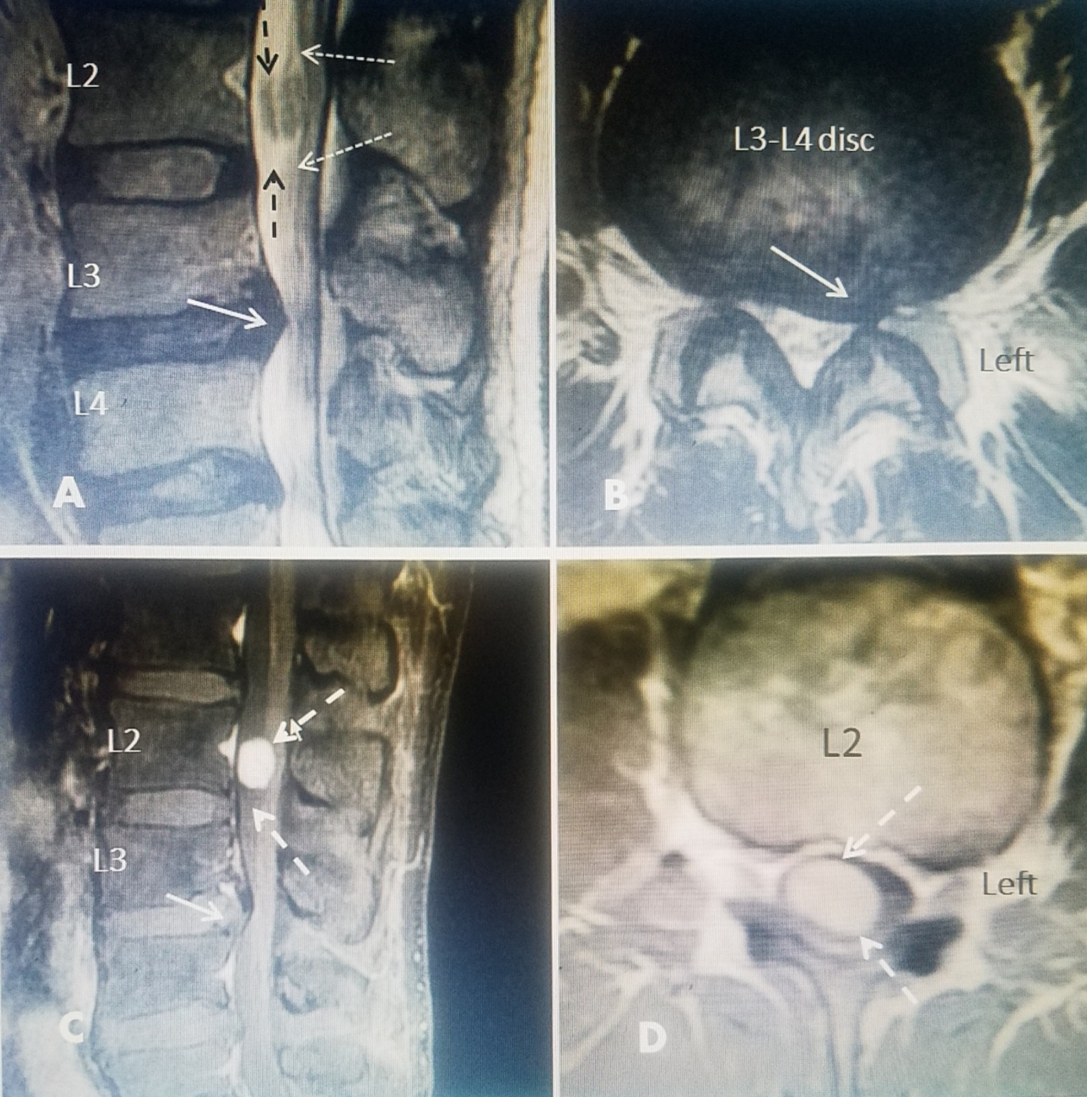
Initial and post-gadolinium (Ga+) magnetic resonance imaging (MRI). A 38-year-old male in previous good health had a minor auto accident. He originally had back pain and then developed tingling and pain down the left leg. No neurologic findings including normal reflex and motor strength. A: Sagittal T2 image showing a definite herniated L3-4 disc (solid white arrow). Report specifically stated L2 normal but there is an intradural oval lesion (dashed black arrows) with apparent dorsal displacement of cauda equina roots (dotted white arrows). B: There was no axial image through L2. Axial image at L3-4 demonstrates a left lateral herniation with foraminal narrowing (solid white arrow). C: A gadolinium (Ga^+^) MRI was performed and the large smooth enhancing lesion at the center of L2 was identified (dashed white arrow). D: Axial Ga^+^ MRI clearly shows the tumor is intradural filling 80% of the canal and more to the right side.

The patient was a healthy 38-year-old male who was involved in a minor automobile accident and was complaining of intermittent back pain that he described as sore and stiff. His pain was 3/10 on visual analog scale. His pain was exacerbated by prolonged sitting and standing, and forward bending. He reported that there were no relieving factors. He was treated with therapy and had the MRI scan six weeks after the accident. It was originally read as a herniated L3-4 disc to the left side. The radiology report specifically stated L2 was normal. He started complaining of left leg pain, primarily in posterior thigh and calf, as well as tingling in the left leg. Because of failure to get symptomatic relief and complaints of tingling in the left leg, he was referred to a neurosurgeon. Neurologic examinations including sphincter function, reflexes, sensory and motor were normal. However, on review of the original MRI scan by the neurosurgeon, a possible intradural lesion was seen at L2 and a stat MRI with gadolinium contrast was ordered revealing a smooth, strongly homogeneously enhancing mass of 14 x 21 x 13 mm in the central and right intradural space behind the L2 vertebral body (Figure 1). It was felt the symptoms were from the disc herniation and the intradural tumor was incidental. He subsequently had transforaminal microdiscectomy at L3-4 for the herniated L3-4 disc with complete resolution of his back and left leg pain and leg tingling. He was explained that the tumor was a separate lesion that would grow over time and was given the option of open laminotomy and tumor resection verse stereotactic radiosurgery. He elected for continued observation of the tumor with follow-up MRI scans and was programmed for stereotactic radiosurgery.

Case 2

**Figure 2:**
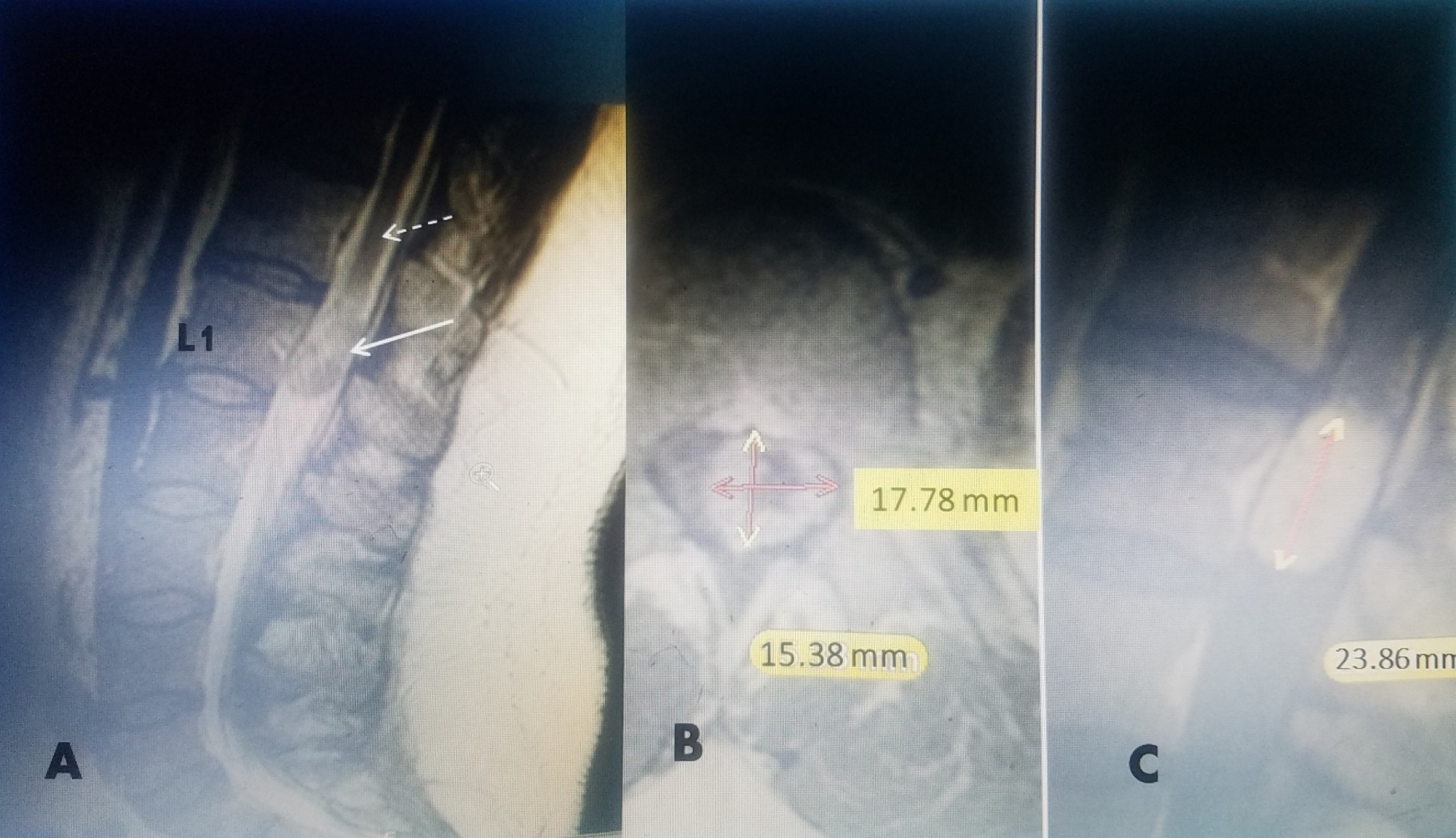
A 45-year-old male with incidental finding of conus medullaris mass. A: T2 sagittal magnetic resonance imaging (MRI) without gadolinium (Ga^+^) showing mixed density expansile mass in conus medullaris. B: Post-Ga^+ ^T1 MRI axial image showing hypodense center with increased peripheral uptake 17.78 millimeters (mm) coronal and 15.38 mm anterior-posterior dimension. C: Post-Ga^+ ^Sagittal T1 with contrast showing isodense 23.86 mm sagittal dimension. Total volume is 6.54 cubic centimeters (cm^3^).

The patient was a healthy 47-year-old male who was involved in a minor automobile accident approximately five weeks prior to presentation. He was the restrained driver of a motor vehicle when it was struck on the driver’s side. He was complaining of constant low back pain that he described as sore, stiff and aching that radiated from the low back into the left hip. His pain was rated a 6-7/10 on a visual analog scale. Sitting and lying down exacerbated his symptoms. His symptoms improved with physical therapy, non-steroidal anti-inflammatory medication and rest. An MRI without gadolinium, of his lumbar spine was performed six weeks after his accident. It was read by the radiologist as a L4-5 posterior central and left paracentral disc herniation, L5-S1 broad-based disc bulge and an expansile mass in the conus medullaris and superior cauda equina. The radiologist recommended that the MRI be repeated with gadolinium contrast. The contrast MRI was compared to the previous MRI which also showed the intradural lesion and he was referred to a neurosurgeon. Neurologic examinations including sphincter function, sensory and motor were normal. His deep tendon reflex was normal except a decreased knee jerk on the right which was 1+/4. On review of the MRI with gadolinium contrast an intradural mass was seen in the conus at L1 measuring 2.4 cm x 1.5 cm with a non-enhancing irregular center and an enhancing periphery (Figure 2). Electromyographic testing and bladder cystometrics both were normal. The patient elected for continued follow-up with serial MRI scans with contrast. He was recommended to have at minimum biopsy and radiosurgery and decided on radiosurgery.

## Discussion

In the two cases presented, coincidentally seen within six weeks of each other, large incidental asymptomatic intradural tumors were found in MRI scans performed after minor automobile accidents. In each case the patient had either new specific minor lumbar pain or radiculopathy leading to the original radiologic study that was being performed for a suspecting lumbar disc. One patient actually did have a 5 mm acute herniated L3-4 disc, lateralized to the side of his pain and numbness but with an incidental intradural tumor at L2, originally missed on the initial MRI examination, while in the other patient the lumbar spine findings did not reveal any disc disease but an incidental mass that was totally asymptomatic within the conus medullaris.

Incidental radiologic findings are defined as any abnormal finding not related to the chief complaint that was the reason for the radiologic study, however it is important that these incidental findings not only be noted by the radiologist but suggested additional films recommended and contact made to the referring physician for both clinical and radiologic follow-up [1,2]. Looking more specifically at the lumbar spine, there are numerous incidental lesions that are found during routine spine examinations. These findings can be divided into extraspinal, vertebral and intraspinal [3-7]. Although the suspicion of spinal pathology, usually disc in origin, is the reason for the radiologic study, there is a relatively large anatomic area surrounding the lumbar spine where there can be numerous extraspinal lesions in the abdomen and retroperitoneal space including the kidneys, aorta, vena cava and iliac vessels as well as pelvic structures, the pre- and paravertebral space, and paraspinal soft tissue and muscles [3,4]. Incidental spinal lesions are divided into intraosseous vertebral spinal lesions identified within the actual vertebrae or disc space distinguishing them from intraspinal lesions [5-7]. The vertebrae are the most common location for incidental findings, comprising 70–80% of incidental spine lesions and commonly include hemangiomas, unsuspected osteoporotic fractures, intraosseous tumors, and unsuspected spinal metastasis [7,8]. Lesions within the spinal canal or neural foramina are defined as intraspinal, are less frequent, but can include migrated disc fragments and commonly include Tarlov cysts, lipomas, tethered cord and thickened filum terminale, dermoid and other developmental tumors and much more infrequently intradural or extradural tumors [9-12]. Besides the obvious location within the spinal canal, differentiating these lesions depends on a combination and comparison using CT reconstruction possible identifying intra-tumoral calcification, existence of adjacent vertebral erosion and detecting enhancement on CT contrast. Classification of intraspinal masses using MRI with and without using Gadolinium contrast can demonstrate patterns of enhancement commonly seen with tumors [8,9]. Included in the radiologic differential diagnosis of an intraspinal mass is always an extruded disc fragment which can migrate within the epidural space and rarely even intradurally but will not have gadolinium enhancement. MRI enhancement patterns can help distinguish neurofibroma and schwannoma, which typically have homogenous and intense enhancement, as in case 1, with a meningioma that typically on MRI has diffuse enhancement. Lumbar ependymoma or myxopapillary ependymoma is most commonly found in the conus medullaris as in case 2 but on MRI imaging tend to be isointense or hypointense on T1 MRI and may have hyperintensity on T2 imaging but with irregular nonhomogeneous enhancement. Ependymomas can also have hemosiderosis in the outer rim, cystic components and ‘ring’ peripheral enhancement. Lipomas are distinguished on high signal intensity T2 and fat imaging. There are reports of numerous other lesions such as dermoid, epidermoids, spinal hemangioblastoma, metastatic tumors and infectious nodules [9-11]. Interestingly, there are several case reports with a similar finding of a symptomatic herniated lumbar disc and an incidental tumor nearby both in the lumbar and cervical spine [12,13]. Finding two such tumors as incidental findings as we report in a short period is unusual. Often intraspinal tumors can have a latent period without symptoms and even when initially symptomatic these tumors can be clinically confused with symptoms of lumbar disc degeneration [12-14]. Review of large surgical series demonstrates the presenting symptoms are a mixture of gradual lumbar pain with and without radiculopathy progressing on to cauda equina syndrome, however, there is commonly a delay in diagnosis since other causes of lumbar pain and radiculopathy are often initially suspected such as herniated disc and spinal degeneration [8,13,14].

In a large series of 1268 cases having lumbar MRI for suspected lumbar disc, 107 or 8.4% had incidental findings. These included in order of frequency fibrolipoma, Tarlov's Cyst and vertebral hemangiomas. There were no tumors in this series [6]. Lumbar and thoracic intradural tumors presenting as incidental, asymptomatic findings, are very uncommon comprising less than 0.05% of large series of incidental MRI studies [8,13]. Spinal tumors comprise 15% of all central nervous system tumors. Their annual incidence is 2-10 per 100,000 and 90% are in patients older than 20 years [8]. Extradural tumors account for 55% of spinal tumors with the most common being cancer metastasis. Intradural tumors account for 45% of spinal tumors. These can be divided into extramedullary (40%) and intramedullary (5%). Lumbar vertebral intradural lumbar tumors make up less than 5% of all spinal tumors. Lumbar tumor types include benign slow growing neuromas and meningiomas involving the nerve roots as well as ependymoma and other neural tumors involving the conus medullaris and cauda equina and schwannomas, meningiomas, filum terminale and conus ependymomas make up 85% of these tumors [8,15,16]. Benign intraspinal tumors found in another series of 14 cases found two schwannomas at L2 and L4-5, a meningioma at L2 and two myxopapillary ependymomas at L3 and L4. Many meningiomas and schwannomas are slow growing and as in these cases the tumor can be quite large yet asymptomatic. In a case report similar to our case, a carcinoid metastasis tumor was incidentally found at L2 with an L4-5 disc causing left sciatica [10].

Treatment for intradural tumors is surgical resection, either through standard laminotomy or more recently using a microsurgical interlaminar approach and more recently spinal stereotactic radiosurgery [8,17-20]. Understanding the growth rate and natural history of these intraspinal tumors is also important. In a study of 23 intradural extramedullary schwannomas, with a five-year follow-up on MRI, 14 of the 23 were in the lumbar area similar to our two cases. The MRI characteristics and gadolinium enhancement pattern and rate of growth were tabulated and it was found that tumors with isodense/high-density and high-intensity and rim enhancement on T2 Gadolinium MRI were smaller with slower growth while high intensity tumors tend to grow significantly faster similar to the first case [18]. Treatment of incidentally found tumors, depending on size, may be initially observation with serial follow-up scans to monitor change in size. The increasing availability of spinal stereotactic radiosurgery makes this a viable option [19,20]. For incidental tumors where the patient does not want open surgery, stereotactic radiosurgery has been shown to be effective in stopping growth and eventual reduction in tumor size over 34 to 36 months [19,20].

## Conclusions

This report of two incidental lumbar intraspinal tumors highlights the importance of careful attention to incidental MRI findings. Careful review of radiologic films and being observant of any incidental findings is critical in detecting uncommon findings like tumors. When the CT or MRI is performed the focus is on 'disc' trauma and 'herniation' incidental findings must be noted by the radiologist or noted later if detected by another physician reviewing the films like in our first case. Although most incidental findings, whether intraosseous or intraspinal, are benign, stable lesions, findings suggestive of vertebral metastatic lesion, osteoporotic fractures and possible intradural tumors require detailed notification and follow-up for proper patient care.
